# Evaluating the Impact of the How‐to Parenting Program on Child Mental Health: A Randomized Controlled Trial in Grade Schools

**DOI:** 10.1111/famp.70081

**Published:** 2025-11-02

**Authors:** Mireille Joussemet, Geneviève A. Mageau, Marie‐Pier Larose, Jean‐Michel Robichaud, Sarah Dufour, Frank Vitaro, Richard Koestner

**Affiliations:** ^1^ Department of Psychology Université de Montréal Montréal Québec Canada; ^2^ School of Public Health Université de Montréal Montréal Québec Canada; ^3^ Inequalities, Interventions and New Welfare State University of Turku Turku Finland; ^4^ Department of Psychology Université de Moncton Moncton Canada; ^5^ School of Psychoeducation Université de Montréal Montréal Québec Canada; ^6^ Department of Psychology McGill University Montréal Québec Canada

**Keywords:** autonomy support, child mental health, How‐to parenting program, program evaluation, randomized controlled trial, school‐aged children

## Abstract

The How‐to Parenting Program teaches parents how to provide autonomy support, structure, and affiliation, the three components of the parenting style shown to be beneficial for children's mental health. Using a waitlist RCT, we assessed its impact on school‐aged children's externalizing and internalizing problems. We also tested whether family composition, participating parents' gender, child age, sex, and baseline mental health modified its effects. Parents (*N* = 293; 80.20% mothers) were randomly assigned to the French version of the 7‐week program or a waitlist condition (i.e., immediate delivery vs. end of study). Parents rated child externalizing and internalizing problems before and after program delivery, as well as 6 and 12 months later. Controlling for unbalanced covariates and baseline levels of problems, multilevel multivariate analyses revealed that compared to the waitlist, the How‐to Parenting Program led to greater decreases in children's externalizing problems immediately after program delivery and that this benefit was sustained over at least 6 months. However, decreases in children's internalizing problems were similar across both conditions. Considering this RCT's methodological strengths (e.g., intent‐to‐treat analyses) and limitations (e.g., intervention diffusion), along with the floor effects inherent to our universal prevention approach, the How‐to Parenting Program's benefits, though small in size, indicate that it could prove an effective public mental health prevention strategy.

## 1 Introduction

The high worldwide prevalence of children's mental health problems is concerning, as they negatively impact their developmental trajectories and life opportunities (Polanczyk et al. 2015). Promotion and prevention programs often target parents, who are seen as key agents in promoting children's health and development (Kaminski et al. 2008; Lindsay and Totsika 2017; Lundahl et al. 2006). While selective and indicated programs are delivered to parents of children who are at risk of displaying problems or already do so, universal parenting programs are offered to any parent (Bayer et al. 2007; Daro and Karter 2019) to shift the entire population distribution toward lower problems (Rose et al. 2008). Allocating resources in this inclusive way can reach families whose difficulties are not very elevated (yet) but who could use some support, while reducing the potential stigma of seeking parenting support. To reach these goals, however, universal parenting programs need to be efficacious in improving children's mental health.

To date, not only have universal parenting programs been less studied than selective and indicated ones, but they have also yielded fewer child mental health benefits (Leijten et al. 2019; Lindsay and Totsika 2017; Salari and Enebrink 2018; Yap et al. 2016), suggesting room for improvement. In the present study, we assessed the efficacy of a popular yet understudied parenting program in improving the mental health of school‐aged children from the general population.

### 1.1 Child Mental Health

During childhood, mental health problems are typically categorized into the two broad externalizing and internalizing categories, which are positively correlated yet distinct (Achenbach 1998; Eisenberg et al. 2000; Krueger 1999). Both externalizing problems (e.g., opposition, aggression, impulsivity) and internalizing ones (e.g., anxiety, depression, social withdrawal) predict later psychopathology and a wide range of undesirable outcomes, such as academic and social difficulties, delinquency, and underemployment (Narusyte et al. 2017; Slemming et al. 2010; Vergunst et al. 2021).

In light of the robust link between parenting quality and child mental health, parenting programs have been proposed as the intervention of choice to foster children's mental health (Bayer et al. 2007; Sanders et al. 2003; Taylor and Biglan 1998). However, we are aware of only six RCTs documenting universal parenting programs' effects on child mental health problems, among which only one targeted parents of school‐aged children. Its results showed that the parenting program under study had no impact on children's externalizing or internalizing problems (Malti et al. 2011), whereas results from the other studies were mixed.^1^

Different reasons may explain these inconsistent findings. A common explanation for a lack of impact lies in potential floor effects (e.g., Hahlweg et al. 2010). Indeed, progress may be more difficult to detect due to minimal levels of problems at pre‐intervention, with reduced room for improvement (Yap et al. 2016). Nonetheless, an additional reason may rest in universal parenting programs' content, as they rarely cover all aspects of the parenting style shown to be strongly related to child mental health (Hoeve et al. 2009; Luyckx et al. 2011; Masten and Shaffer 2006; Yap and Jorm 2015).

### 1.2 Parenting

Parenting studies have repeatedly suggested that a combination of three components constitutes the most favorable parenting style (Gray and Steinberg 1999; Grolnick et al. 1997; Schaefer 1965) for child mental health (Dwairy et al. 2010; Lavrič and Naterer 2020), often called authoritative (Baumrind 1966, 1971). These three key components are (a) affiliation, (b) structure, and (c) autonomy support (AS; Aunola and Nurmi 2005). Indeed, positive child outcomes are fostered the more parents (a) are involved, caring, and accepting; (b) provide an organized environment; and (c) are empathic, take their children's experiences into account, and support their volitional functioning. This knowledge emerged from developmental (e.g., Gray and Steinberg 1999) and motivational (e.g., Grolnick et al. 1997; Ryan and Deci 2017) psychology. Importantly, each of these parenting components is uniquely associated with better child mental health, whereas their opposites (e.g., rejecting, chaotic, and controlling practices) have been linked to more psychological symptoms (Rohner and Britner 2002; Valiente et al. 2007).

A similar pattern of findings emerges from applied research. Indeed, a meta‐analysis examining the content of various parenting programs found that the components that predicted child benefits to a larger extent were positive interactions, consistent responding, and emotional/empathic communication (akin to affiliation, structure, and AS, respectively; Kaminski et al. 2008). Basic and applied studies thus suggest that parenting programs addressing all three positive parenting components should be well‐suited to foster child mental health, perhaps especially when delivered at the universal level.

We aimed to assess the impact of an accessible parenting program that includes all three key parenting components on child mental health. According to Joussemet et al. (2014), the *How to talk so kids will listen & listen so kids will talk* program (called the “How‐to Parenting Program” herein; Faber and Mazlish 1995) translates the essence of AS into readily applicable skills in addition to helping parents convey an unconditional bond and provide clear structure. Indeed, the How‐to Parenting Program is based on Ginott's (1965) communicative approach to parenting, whose writings (Ginott 1959, 1965) on impersonal and empathic limit‐setting also inspired the operationalization of AS (Koestner et al. 1984) in the self‐determination theory (SDT) framework (Deci and Ryan 2000; Ryan and Deci 2017). In SDT, AS does not refer to promoting independence (Soenens et al. 2007), as relatedness, competence, and autonomy (i.e., volition) are posited as three essential psychological needs. Parents can support their children's autonomy by acknowledging and validating their feelings, explaining requests and limits' rationales, and promoting children's active participation in problem‐solving and developmentally appropriate decision‐making (Mageau and Joussemet 2022).

In a pre‐post evaluation of the How‐to Parenting Program, parents of school‐aged children reported improvements in all three key parenting components as well as decreases in their children's internalizing and externalizing problems (Joussemet et al. 2014), which were sustained the following year (Mageau, Joussemet, Paquin, et al. 2022). Next, in a waitlist RCT conducted with parents of school‐aged children, the How‐to Parenting Program was found to lead to greater AS, an improvement that was maintained over 1 year. It also helped foster affiliation (and potentially structure) among parents with initially lower scores on these components (Mageau, Joussemet, Robichaud, et al. 2022). This RCT (Joussemet et al. 2018) also aimed to assess this program's impact on child mental health, the more distal yet ultimate outcome of parenting programs. Moreover, while we would expect that benefits would not be limited to a subgroup of children, some could benefit more than others, depending on the initial problem levels or certain sociodemographic characteristics. Exploring potential moderators typically tested in parenting program evaluations (e.g., Gardner et al. 2010) would thus seem warranted.

## 2 The Present Study's Objectives and Hypotheses

We conducted an RCT to assess the impact of the How‐to Parenting Program on school‐aged children's mental health, using the same sample and design described in (Mageau, Joussemet, Robichaud, et al. 2022). The protocol of this efficacy trial was preregistered in a primary clinical trial registry (ClinicalTrials.gov NCT03030352; see Joussemet et al. 2018). We aimed to compare, over 1 year, the mental health problems (i.e., externalizing and internalizing behaviors) of children whose parents were offered the program to those of children whose parents were assigned to a waitlist (WL). Our secondary goal was to explore whether the program's efficacy (or lack thereof) was moderated by (a) baseline levels of child mental health, (b) child age, (c) child sex, (d) parent gender, and (e) family composition (two‐ vs. one‐parent families).

We predicted that children of parents assigned to the How‐to Parenting Program would demonstrate fewer externalizing and internalizing problems at post‐intervention compared to children of parents on the waitlist, controlling for their respective baselines, and that these expected program benefits would be sustained over 1 year. We had no hypothesis about potential effect modifiers, as this is the first RCT assessing the impact of the How‐to Parenting Program on child mental health. We followed the CONSORT standard guidelines for social and psychological interventions when describing our Method and Results (Montgomery et al. 2018).

## 3 Method

### 3.1 Design and Procedure

We conducted a waitlist RCT in 15 French‐speaking elementary schools in the large and pluricultural city of Montréal, in the province of Québec (Canada). The study was approved by the Ethical Research Committee of the Université de Montréal. Detailed procedure information can be found in Joussemet et al. (2018), Mageau, Joussemet, Robichaud, et al. (2022), and Lafontaine et al. (2025). Participants from 4 to 6 schools/year were recruited over three yearly waves. All parents within each school received a pamphlet advertising the study and inviting them to an information session. During that session, the study and the program's format were detailed. The program's name was also revealed, though the content of the program was described in very general terms, and none of its anticipated benefits were mentioned. Interested parents completed an informed consent form and the pre‐intervention questionnaire at the end of this information session.

The only inclusion criteria for participating parents were to have at least one child attending one of the participating grade schools and be able to attend a parenting program delivered in French, the language spoken by the majority in the city. To avoid introducing a bias by letting parents choose their target child, we asked them to select the child who was at least 8 years of age and closest to age 9 (i.e., about the midpoint of the age range).

Randomized allocation was made within each school. Participating parents completed their pre‐intervention assessment (T1; paper‐pencil) before being randomly assigned with a 1:1 ratio to either the experimental How‐to condition (i.e., being offered the program in 2 weeks) or the waitlist (WL) control condition (i.e., being offered the program in 14 months, at the end of the study). Parents gave their completed T1 questionnaire to the research assistant in a sealed blank envelope upon leaving the information session. All anonymous envelopes from each school were returned to the research coordinator, who shuffled them and randomly split them into two piles within each school, corresponding to the experimental and WL conditions. Each parent was then assigned an identification number, which was used to match follow‐up questionnaires.

**FIGURE 1:**
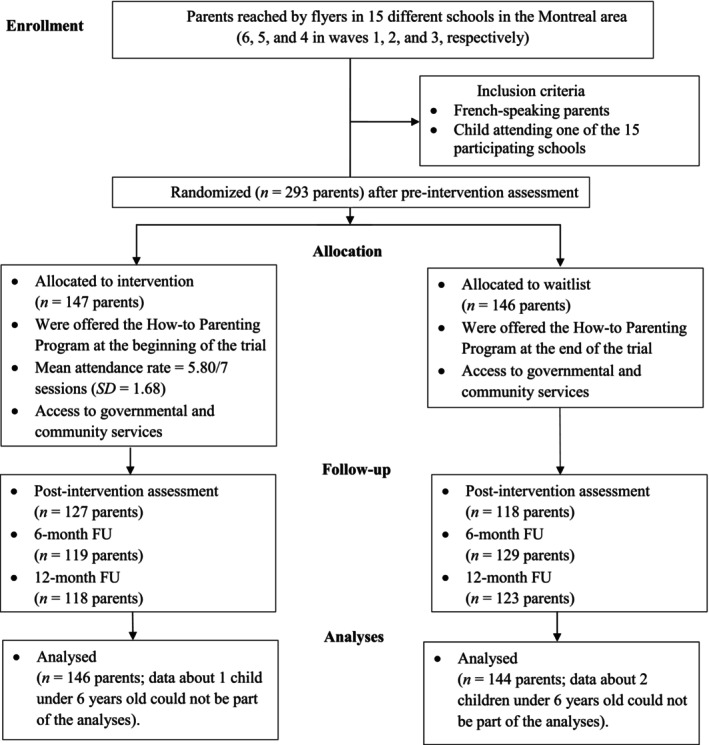
Flow chart.

The How‐to Parenting Program was delivered in children's schools for 7 consecutive weeks, from 7:00 to approximately 9:30 p.m. One week after the end of program delivery to parents in the experimental condition, all parents were invited to complete the post‐intervention questionnaire (T2; paper format or online, according to parents' preference). Six and 12 months following T2, all parents were invited to complete follow‐up (FU) assessments (T3 and T4, paper format or online). Figure 1 displays the study's flowchart and its different assessments.

### 3.2 Participants

The RCT comprised 293 parents (147 parents in the How‐to condition and 146 parents in the WL control condition; 293 families). There was an experimental and a WL group within each of the 15 participating schools (a total of 30 parenting groups, 15 per condition), and 5–14 parents per group. When two parents of a family took part in the program (10 families in the How‐to condition and 11 parents in the WL control condition), both were asked to complete questionnaires, but data from solely one parent per dyad were kept, which was selected randomly. Given the final sample size and according to our previous power calculation (Joussemet et al. 2018), we expected sufficient power to detect effects of small to moderate sizes.

Aligned with the universal prevention approach, all parents from each participating grade school were invited (no inclusion/exclusion criteria other than language). The number of eligible children per participating school varied from 333 to 662 children (*M* = 457), such that a little more than 7000 fliers were sent to families via children's school bags. Therefore, we estimate that about 4% of contacted families had a parent who took part in the RCT.

A large proportion of participating parents reported that their targeted child was experiencing some difficulties, a common pattern (e.g., Bodenmann et al. 2008), as parents tend to self‐select into programs based on their children's risk (Dadds and Roth 2008). Indeed, though no children had a score falling in the Clinical range of the Child Behavior Checklist (CBCL; Achenbach and Rescorla 2001), a little more than a third of children's internalizing and/or externalizing CBCL scores fell in the Borderline range at pre‐intervention, which is about five times more than in the general population (Achenbach and Rescorla 2001).

Table 1 presents the sample's sociodemographic characteristics, while Table 2 presents correlations between the study's main variables. About two‐thirds of parents (67.13%) identified themselves as Canadian, White, or French. The others identified themselves as Arabic (7.07%), Haitian (3.18%), or Hispanic (3.13%) or reported one of 34 other ethnicities (19.49%). Parents who participated in the study were mainly in a two‐parent family (86%), a higher rate than the provincial rate of 73.5% (Lafontaine et al. 2025). Their socioeconomic status was elevated, especially in terms of education, as 74.31% of parents had a university diploma (vs. 31% in the province; Ministère de la Famille 2021). In terms of familial income, the median familial income reported by participants fell in the $CAN50,000 to $CAN75,000 range, which is similar to the provincial median of $CAN52,519 (Lafontaine et al. 2025).

**TABLE 1:** Characteristics of the sample by experimental conditions, at pre‐intervention.

Socio‐demographic characteristics		Conditions		
	Full sample	Experimental	Control	*p*
		(How‐to)	(Waitlist)	
	*N =* 293	*N =* 147	*N =* 146	
**Categorical variables, *N* (%)**				
Child sex (boys)	145 (50.0)	75 (51.4)	70 (48.6)	0.64
	*N = 290*	*N = 146*	*N = 144*	
Parent gender (men)	58 (19.8)	34 (23.1)	24 (16.4)	0.15
Family income				0.05
Less than 15,000$	38 (13.3)	25 (17.2)	13 (9.3)	
Between 15,000$ and 30,000$	36 (12.6)	20 (13.8)	16 (11.4)	
Between 30,000$ and 50,000$	56 (19.6)	30 (20.7)	26 (18.6)	
Between 50,000$ and 75,000$	55 (19.3)	19 (13.1)	36 (25.7)	
Between 75,000$ and 100,000$	100 (35.1)	51 (35.2)	49 (35.0)	
	*N = 285*	*N = 145*	*N = 140*	
Education				0.90
High school or less	13 (4.5)	6 (4.2)	7 (4.9)	
College or professional training	61 (21.2)	33 (22.9)	28 (19.4)	
Undergraduate university diploma	125 (43.4)	62 (43.1)	63 (43.8)	
Graduate university diploma	89 (30.9)	43 (29.9)	46 (31.9)	
	*N = 288*	*N = 144*	*N = 144*	
Family composition				0.18
Two‐parent family	246 (86.0)	120 (83.3)	126 (88.7)	
One‐parent family	40 (14.0)	24 (16.7)	16 (11.3)	
	*N = 285*	*N = 144*	*N = 142*	
**Continuous variables, Mean (SD)**				
Child age	7.6 (1.92)	7.69 (1.91)	7.51 (1.94)	0.18
	*N = 291*	*N = 147*	*N = 144*	
Parent age	40.26 (5.76)	39.39 (5.74)	41.13 (5.66)	0.01
	*N = 285*	*N = 142*	*N = 143*	
AT parenting (CR)	1.92 (0.60)	2.04 (0.65)	1.79 (0.52)	0.03
	*N = 108*	*N = 55*	*N = 53*	
AS parenting (PR)	5.49 (0.72)	5.42 (0.72)	5.56 (0.71)	0.09
	*N = 289*	*N = 146*	*N = 143*	

Abbreviations: AS, autonomy‐supportive; AT, autonomy‐thwarting; CR, child‐reported; PR, parent‐reported.

**TABLE 2:** Intercorrelation among the study's main variables.

Variables		1	2	3	4	5	6	7	8	9	10	11	12
1.	Parent age	1											
2.	Family income	0.12*	1										
3.	T1 AT parenting (CR)	0.04	−0.28*	1									
4.	T1 AS parenting (PR)	0.04	0.17*	−0.22*	1								
5.	T1 Externalizing behaviors	−0.07	0.07	0.08	−0.18*	1							
6.	T2 Externalizing behaviors	−0.04	0.09	0.00	−0.12	0.74*	1						
7.	T3 Externalizing behaviors	−0.06	0.03	0.07	−0.14*	0.68*	0.72*	1					
8.	T4 Externalizing behaviors	0.01	0.03	0.01	−0.12	0.61*	0.76*	0.71*	1				
9.	T1 Internalizing behaviors	0.04	0.07	0.19*	−0.17*	0.51*	0.39*	0.44*	0.28*	1			
10.	T2 Internalizing behaviors	0.02	0.09	0.04	−0.05	0.42*	0.62*	0.49*	0.45*	0.69*	1		
11.	T3 Internalizing behaviors	−0.02	0.00	0.09	−0.09	0.38*	0.47*	0.68*	0.46*	0.63*	0.73*	1	
12.	T4 Internalizing behaviors	0.04	0.04	0.05	−0.05	0.33*	0.50*	0.47*	0.66*	0.51*	0.68*	0.68*	1

*Note:* T1 = pre‐intervention; T2 = post‐intervention; T3 = 6‐month follow‐up; T4 = 1‐year follow‐up.

Abbreviations: AS, autonomy‐supportive; AT, autonomy‐thwarting; CR, child‐reported; PR, parent‐reported.

* *p* < 0.05.

One out of five participating parents was a father (19.8%), and half of the targeted children were girls. In terms of children's ages, 50.86% were between 5 and 7 years old, and 39.86% were between 8 and 10. The remainder (8.25%) were 11 and 12 years old or, unexpectedly and probably due to parents' misunderstanding of inclusion criteria, 3 or 4 years old (1.02%). In the present study, we excluded the latter three children from further analysis, as the CBCL/6–18 is not an appropriate tool to assess preschoolers' mental health.

### 3.3 Intervention

The How‐to Parenting Program is a manualized parenting program developed by Faber and Mazlish in Long Island (NY, USA) to accompany their popular *How to talk so kids will listen & listen so kids will talk* book (the How‐to book herein; Faber and Mazlish 1980). The original workshop can be easily delivered to English‐speaking parents via a DVD or CD kit (Faber and Mazlish 1995). However, in the present RCT, the program was delivered in French by dyads of trained facilitators who relied entirely on written material (Faber and Mazlish 2001a; see Lafontaine et al. 2025, for more material details). Editors of the French material translated the material without adaptation.

All parents were offered a copy of the translated How‐to book (Faber and Mazlish 2002) for weekly readings and a workbook to complete exercises during and between sessions (Faber and Mazlish 2001b). The program's first six (topical) sessions correspond to the book's main chapters (i.e., helping children deal with their feelings, engaging cooperation, alternatives to punishments, encouraging autonomy, descriptive praise, and freeing children from playing roles), whereas the last one is integrative. This program teaches a total of 30 concrete parenting skills (see Table 3).

**TABLE 3:** Skills taught in the How‐to Parenting Program.

Session/Chapter title		Skills	Examples
Session 1**/** Chapter 1	Helping children deal with their feelings	Listen to him/her with full attention; Acknowledge with a word, and/or a sound; Try to name the child's feeling; Give him/her what s/he desires in fantasy	Look at the child when s/he speaks. “Oh…” ; “Hm”; “That can feel scary” “I wish I could make a snack appear for you right now”
Session 2**/** Chapter 2	Engaging cooperation	Describe what the problem is; Provide some more information; Remind the child with just one word; Express your own feelings without attacking the child's character; Write a note	“There are boots in the middle of the hallway” “It's hard to walk when boots are blocking the way and wetting the floor” “Kids, the boots” “I feel irritated when I come back home and can't walk in the hallway” “Please bring us back on our rack” *(signed: your boots)*
Session 3**/** Chapter 3	Alternatives to punishments	Express own feelings without attacking the child's character; State your expectation; Show him/her how to make amends; Give him/her a two options; Take action if needed; Problem‐solve with child	“I don't like to see food residues on the couch” “I expect eating to take place in the kitchen” “This couch needs to be cleaned. Here's a wet sponge with some soap on it” “You can either eat your snack in the kitchen before watching tv or watch tv without a snack” After giving the choice (see above), take away the snack. Acknowledge child's feelings; express yours; brainstorm (write child's ideas and your own); Select one idea, plan and implement it.
Session 4**/** Chapter 4	Encouraging autonomy	Let him/her decide; Respect the child's struggle; Limit the number of your questions; Don't rush to answer his/her questions; Promote some outside resources; Don't take away the child's hope.	“Do you want the blue or the red shirt?” “Pouring milk in a glass can be tricky, sometimes it helps to use a wide glass” Let child talk about his/her day when s/he wants to. “Interesting, why do *you* think kids lose their teeth?” “I wonder what the dentist would say” “An astronaut, what an interesting career.”
Session 5**/** Chapter 5	Descriptive praise	Describe the child's behavior or accomplishment; Describe own feelings; Summarize the child's behavior with a noun.	“I see toys on their shelf” “It feels good to sit on the couch easily” “That's what I call *organization*”
Session 6**/** Chapter 6	Freeing children from playing roles	Notice counter role behavior from the child; Provide him/her with counter role opportunities; Let the child overhear positive comments; Model appropriate behavior; Recall one of the child's counter role behavior in the past; If s/he reverts to an old role, state your feeling and expectation.	Example: the “sore loser” “You shook the winner's hand” “Let's play a game of …” “Suzie congratulated me when …” “Congratulations for winning this game!” “I remember when you congratulated me for winning at …” “I expect you to congratulate the winner after a match”
Session 7	Integration	Open, guided discussion; Activity about managing typical parent‐child interactions integrating various skills; Description of participants' accomplishments in learning skills.	

*Note:* Retrieved from Joussemet et al. (2018).

In a typical session, the first 20 min are allotted to discuss the implementation of the skills presented the previous week (challenges as welcome as successes) and/or readings. Next, a perspective‐taking exercise is used to introduce the current session's main theme, notably by helping parents experience how children may feel when hearing common, yet suboptimal utterances. Alternative skills (an average of 5 skills per week) are then presented through comic strips illustrating parent–child interactions. The remainder of the sessions consists of role‐playing exercises and structured discussions to help parents learn these alternative skills. Finally, facilitators introduce homework and remind parents of the importance of trying to put skills into practice. During the last session, parents brainstorm together about some challenging situations to identify which skills could prove useful. They also receive a certificate and a list summarizing the program's skills.

Copyright owners of the curriculum do not require specific training or minimal qualifications to deliver the How‐to Parenting Program. However, in the present RCT, all facilitators (graduate students in psychology, parents, or adults involved in an education‐related domain) took part in a 3‐day training led by a mentor with decades of experience offering this program in French. During this training, facilitators learned about the program's content by taking part in it as parents would. They also learned about the importance of content fidelity as well as their expected role and posture (e.g., modeling the program's skills, avoiding acting as an expert, and conveying unconditional regard). In each dyad, at least one of the facilitators had already delivered the program in the past. After each session, co‐facilitators were encouraged to debrief with one another and, if needed, to consult the first author, also a licensed psychologist.

### 3.4 Measures

At T1, parents provided sociodemographic information by answering questions about their age, gender, education level, family composition (two‐ or one‐parent family), and annual familial income, as well as their targeted child's age and sex (see Table 1).

As part of this RCT, parents completed various parenting and mental health measures. Children were also invited to fill out questionnaires on those outcomes, if they were old enough to do so (at least 8 years old; see Joussemet et al. 2018, for more details). As reported in (Mageau, Joussemet, Robichaud, et al. 2022), two parenting variables were found to be unbalanced across experimental conditions at T1 despite randomization and are thus covariates in the present study. These are parent‐reported attitudes toward AS, assessed with the Parental Attitude Scale (*α* = 0.70–0.75; Gurland and Grolnick 2005) and child‐reports of autonomy‐thwarting (AT), assessed with the Perceived Parental Autonomy Support Scale (*N* = 112; *α* = 0.73 to 0.84; Mageau et al. 2015).

#### 3.4.1 Child Behavior Problems

At each assessment time (T1–T4), parents evaluated their targeted child's mental health using the Child Behavior Checklist (CBCL; Achenbach and Rescorla 2001). The CBCL is one of the most widely used validated instruments to assess child mental health. This instrument clusters mental health in two broad subscales—externalizing and internalizing problems. The externalizing syndrome (35 items) reflects rule‐breaking, oppositional, and aggressive behaviors, whereas the internalizing syndrome (32 items) reflects anxiety, withdrawal, depression, and somatic problems. All behaviors are assessed as occurring either *never* (0), *sometimes* (1), or *often* (2). Reliability coefficients for the externalizing and internalizing subscales were excellent at all assessment times (*α*_T1–T4_ ranging from 0.90 to 0.91 and from 0.88 to 0.91, respectively).

As any missing answer results in a missing raw score when sums are calculated, we used average‐item scores to reduce the number of missing values and their related biases in the main analyses. When missing data were present for eight or fewer items, these items received the score of 0 as per the CBCL scoring procedure. Since no parent skipped more than 20 items, all CBCL could be considered valid. We also multiplied the CBCL scores by 100 before the main analyses to facilitate the convergence of the multilevel models (theoretical range = 0–200), given that Mplus rounds parameter estimates to the third decimal. The sum scores were, however, used for supplemental descriptive analyses to provide practically useful information about problem behavior changes.

#### 3.4.2 Fidelity of Program Delivery

To assess all aspects of program fidelity (Dane and Schneider 1998), group facilitators audiotaped their sessions and information was provided by parents and facilitators (e.g., enthusiasm, attendance, book reading). At T4, parents assigned to the WL condition were asked if they had read the How‐to book and to what extent (i.e., *none*, *less than half*, *half*, *most of*, or *all of* the book).

### 3.5 Plan of Analyses

#### 3.5.1 Preliminary Analyses

##### 3.5.1.1 Randomization

Randomization success for this RCT was previously tested among 15 key sociodemographic and parenting variables (Mageau, Joussemet, Robichaud, et al. 2022). Using a liberal critical *p*‐value of 0.10, a univariate approach identified unbalanced variables. All variables that were unbalanced between conditions were kept as covariates in the present study's main analyses (i.e., parental attitudes toward AS, child perceptions of AT, parent age, and family income). We then added pre‐intervention levels of problem behaviors as additional covariates to ensure that condition differences in problem behaviors were independent of baseline levels.

##### 3.5.1.2 Fidelity

The fidelity with which the How‐to Parenting Program was delivered during this RCT is fully described in Lafontaine et al. (2025); we only briefly summarize some key information in the preliminary results section.

##### 3.5.1.3 Attrition

We examined attrition patterns in both conditions. To do so, we conducted binomial logistic regressions to verify if baseline characteristics were associated with the probability of missing one or more assessment time points. The percentage of participants with missing data on study variables can be found in Table S1.

#### 3.5.2 Main Analyses

We conducted multivariate, multilevel analyses with the MLR estimator in Mplus. We chose these analyses because they allow for non‐normal and missing data and because they examine differences between conditions while considering the non‐independence of the multiple data points nested within each participant and any unbalanced variables at baseline.

Therefore, all participants were included in the main analyses, no matter the number of questionnaires they completed, as missing data were handled using full information maximum likelihood (FIML; Larsen 2011).

Unbalanced covariates and baseline levels of problems were controlled in all our regression models. As previously mentioned, data from solely one parent per family were included in the analyses. Finally, we used an intent‐to‐treat approach to increase the findings' external validity. All participants were thus retained in all analyses, regardless of the extent to which they missed program sessions (Newell 1992).

##### 3.5.2.1 Experimental Manipulation's Short‐Term Impact

We estimated, at the within‐person level, the slopes of behavioral problems such that their intercepts would represent participants' post‐intervention (T2) ratings, and we examined the impact of the How‐to Parenting Program on internalizing and externalizing problem behaviors by regressing these intercepts and slopes on the condition variable (0 = WL condition; 1 = How‐to condition), identified covariates (all centered on their grand mean), and the outcome's pre‐intervention level.

The model can be summarized with the following equations. Externalizing and internalizing behaviors were estimated within the same model, and their covariance was accounted for, but we present levels 1 and 2 equations separately for the sake of simplicity. *γ*_00_ is the fixed effect of the intercept, while *γ*_10_ is the fixed effect of the slope. *U*_0j_ is the random intercept, while *e*_*ij*_ is the participant residual variance (PR = parent report; CR = child report).

Level 1:

Externalizing behaviors_*ij*_ = *β*_0_ + *β*_1_Time_*ij*_ + *e*_*ij*_

Level 2:

*β*_0_ = *γ*_00_ + *γ*_01_Condition_*j*_ + *γ*_02_Pre‐intervention levels of externalizing behaviors_*j*_ + *γ*_03_Parental income_j_ + *γ*_04_Parental age + *γ*_05_AS parenting (PR)_*j*_ + *γ*_06_AT parenting (CR)_j_ + *U*_0*j*_

*β*_1_ = *γ*_10_ + *γ*_11_Condition_*j*_ + *U*_1*j*_Level 1:

Internalizing behaviors_*ij*_ = *β*_0_ + *β*_1_Time_ij_ + *e*_*ij*_

Level 2:

*β*_0_ = *γ*_00_ + *γ*_01_Condition_j_ + *γ*_02_Pre‐intervention levels of internalizing behaviors_*j*_ + *γ*_03_Parental income_*j*_ + *γ*_04_Parental age + *γ*_05_AS parenting (PR)_*j*_ + *γ*_06_AT parenting (CR)_*j*_ + *U*_0*j*_

*β*_1_ = *γ*_10_ + *γ*_11_Condition_*j*_ + *U*_1*j*_

To quantify the efficacy of the program, the size of these main analyses' effects was determined using Cohen's *f* ^2^ for multilevel models (Lorah 2018). According to Cohen (1992), *f* ^2^ values of 0.02, 0.15, and 0.35 are considered small, medium, and large, respectively. To compare our results with previous studies, we also computed Cohen's *d* effect sizes (where 0.20 is considered small, 0.50 is considered medium‐sized, and 0.80 is large) at T2 using path analysis, where T2 behavior problems were regressed on the condition variable, the previously identified covariates, and the baseline score.

##### 3.5.2.2 Impact's Stability

We tested whether the effects detected post‐intervention (or lack thereof) remained stable over the following year. To do so, we modeled curvilinear and linear mental health trends from T2 to T4. Non‐significant between‐condition differences (condition on slopes) implied that children in both conditions had similar behavior problem trajectories from T2 to T4. A non‐significant within‐condition difference (slope) implied that the child's mental health remained stable from T2 to T4 in that condition.

##### 3.5.2.3 Moderation

We explored whether the effect (or lack thereof) of the How‐to Parenting Program at T2 was moderated by (a) baseline levels of child mental health (externalizing and internalizing problems), (b) child age, (c) child sex, (d) parent gender, and (e) family composition. We tested these five models separately (i.e., one per potential moderator).

##### 3.5.2.4 Within‐Condition Changes

We examined how children's externalizing and internalizing symptoms within each condition changed from pre‐intervention (T1) to post‐intervention (T2) and each follow‐up assessment (T3 and T4). To do so, we conducted multilevel multigroup analyses (within each condition, modeled as one group). At the within‐person level, we regressed behavior problem assessments on dummy codes representing the differences between T1 and subsequent assessments (T2, T3, and T4) and estimated the intercepts (scores at T1) and slopes for each dummy code (differences from T1 to T2, from T1 to T3, and from T1 to T4), while allowing them to covary.

## 4 Results

### 4.1 Preliminary Analyses

#### 4.1.1 Randomization

Randomization was generally successful, as child behavior problems at T1 were similar across conditions (all *p*s ≥ 0.82), and only four variables, previously reported in (Mageau, Joussemet, Robichaud, et al. 2022), were unbalanced between conditions. Compared to the WL condition, parents in the How‐to condition were younger (*p* = 0.01), tended to have a lower family income (*p* = 0.07), tended to report lower AS at T1 (*p* = 0.10), and were rated by their targeted child as more autonomy‐thwarting (*p* = 0.03). As these differences remained significant at the multivariate level (all *p*s ≤ 0.10), these variables were kept as covariates in later analysis. Raw scores of child behavior problems in each condition at all time points are presented in Table 4.

**TABLE 4:** Raw means of children‘s problem behaviors and raw proportions of scores falling in the borderline range.

	Mean (SD)			
	*N* (%) Borderline level			
	*N* total			
	Pre‐intervention	Post‐intervention	6‐month FU	1‐year FU
	(T1)	(T2)	(T3)	(T4)
	Externalizing behaviors			
Condition				
Experimental (How‐to)	0.29 (0.21)	0.19 (0.19)	0.18 (0.22)	0.18 (0.19)
	52 (36.9%)	21 (16.7%)	20 (16.8%)	20 (17.2%)
	141	126	119	116
WL control				
	0.29 (0.22)	0.23 (0.20)	0.23 (0.18)	0.21 (0.18)
	52 (37.4%)	28 (23.7%)	32 (25.4%)	26 (21.7%)
	139	118	126	120
	Internalizing behaviors			
Condition				
Experimental (How‐to)	0.28 (0.23)	0.19 (0.20)	0.20 (0.18)	0.17 (0.19)
	53 (37.6%)	28 (23.1%)	26 (23.2%)	26 (22.8%)
	141	121	112	114
WL control				
	0.27 (0.22)	0.19 (0.18)	0.18 (0.22)	0.19 (0.18)
	46 (34.1%)	17 (15.7%)	24 (20.7%)	26 (23.0%)
	135	108	116	113

Abbreviations: FU, follow‐up; WL, waitlist; SD, standard deviation.

#### 4.1.2 Fidelity

The RCT followed its pre‐registered planned design (Joussemet et al. 2018) with only three exceptions. Specifically, compared to the planned RCT, the actual one included a larger number of participants (293 vs. 256 parents), a smaller number of participating schools (15 vs. 16), and one fewer wave of recruitment (3 vs. 4).

All facilitators delivered the full program to their group. Content fidelity and exposure were high, with 87% of the program's planned activities being coded as fully delivered by independent coders (ICC = 0.79), and less than 30% of parents missing more than one session. However, differentiation was imperfect, as 20 WL parents (13.7%) reported having read at least some of the How‐to book, and 47 (32.2%) did not answer the reading question (see Lafontaine et al. 2025, for more detailed fidelity information). Among WL parents who reported reading the How‐to book, none read it entirely, but four read most of it, three read half of it, and 13 read less than half of it.

#### 4.1.3 Attrition

As reported by Mageau, Joussemet, Robichaud, et al. (2022), there was no differential attrition in this RCT. Parents who completed questionnaires at each of the four timepoints did not differ from those who missed at least one data collection timepoint on a pool of nine variables (condition and eight sociodemographic characteristics; all *p*s ≥ 0.674).

### 4.2 Main Analyses

#### 4.2.1 Short‐Term Impact on Child Mental Health and Impact's Stability

A multivariate, multilevel model adjusted for identified covariates (i.e., parental age, family income, T1 parent‐reported AS, and child‐reported AT) and for pre‐intervention level of the outcome (Table 5) revealed that children in the How‐to condition had lower levels of *externalizing* problems than children in the WL condition at post‐intervention (T2; *β* = −3.39, *p* = 0.03, *f* ^2^ = 0.02; *d* = −0.21).

**TABLE 5:** Multilevel linear model testing the impact of the experimental manipulation on children's problem behaviors.

Externalizing behaviors	*β*	SE	*p*
Intercept (T2)	23.04	1.10	< 0.01
Experimental condition^a^	−3.39	1.54	0.03
Parent Age	−0.04	0.14	0.76
Family income	−0.17	0.64	0.80
T1 AT parenting (CR)	0.89	2.39	0.72
T1 AS parenting (PR)	0.19	1.10	0.87
T1 Externalizing behaviors	0.64	0.03	< 0.01
Slope (from T2 to T4)	−0.17	0.10	0.08
Experimental condition^a^	0.06	0.15	0.70
Parent Age	0.01	0.01	0.40
Family income	−0.01	0.05	0.82
T1 AT parenting (CR)	−0.01	0.16	0.95
T1 AS parenting (PR)	−0.09	0.10	0.36
T1 Externalizing behaviors	−0.01	0.01	< 0.01

Internalizing behaviors	*β*	SE	*p*
Intercept (T2)	19.82	1.18	< 0.01
Experimental condition^a^	−0.82	1.71	0.63
Parent Age	−0.02	0.16	0.91
Family income	−0.07	0.63	0.91
T1 AT parenting (CR)	−1.01	2.89	0.73
T1 AS parenting (PR)	1.11	1.31	0.40
T1 Internalizing behaviors	0.56	0.04	< 0.01
Slope (from T2 to T4)	0.01	0.11	0.98
Experimental condition^a^	−0.08	0.17	0.64
Parent age	−0.01	0.01	0.73
Family income	−0.01	0.06	0.91
T1 AT parenting (CR)	0.08	0.17	0.64
T1 AS parenting (PR)	−0.09	0.10	0.42
T1 Internalizing behaviors	−0.01	0.01	< 0.01

*Note:* T1 = pre‐intervention; T2 = post‐intervention; T3 = 6‐month follow‐up; T4 = 1‐year follow‐up.

Abbreviations: AS, autonomy‐supportive; AT, autonomy‐thwarting; CR, child‐reported; PR, parent‐reported.

^a^ Waitlist control condition [0] vs. How‐to condition [1].

**FIGURE 2:**
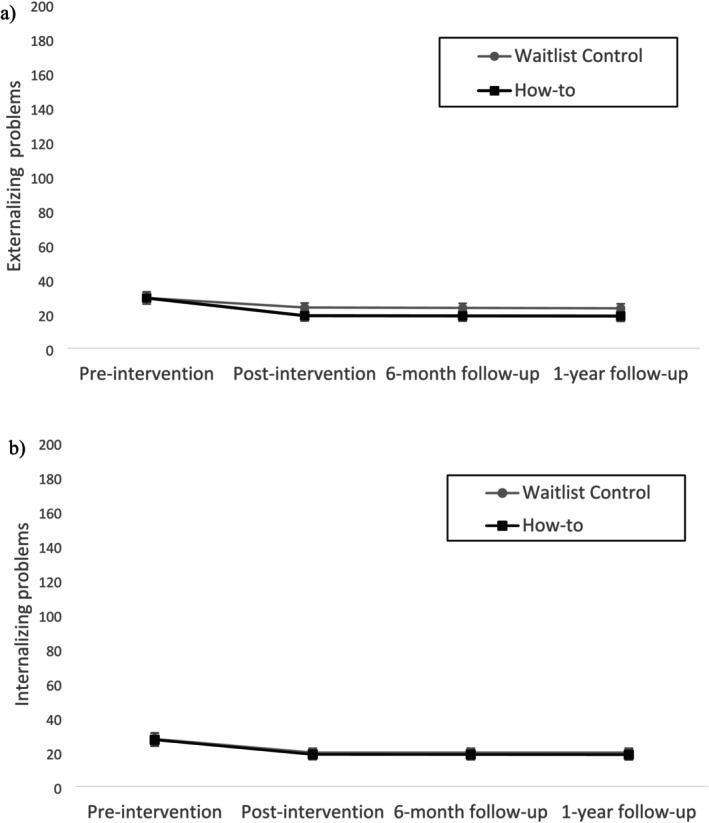
Children's (a) externalizing and (b) internalizing problem behaviors by condition. CBCL scores were multiplied by 100 to facilitate convergence in the MPlus software.

Examining the stability of this effect from T2 to T4 revealed the absence of any curvilinear and linear trend difference between conditions, thereby implying that the observed condition differences at T2 remained stable over the next year (condition on both curvilinear and linear trends had *p*s ≥ 0.08; see Figure 2a), and within conditions, thereby implying that externalizing problems at T2 remained stable from T2 to the 1‐year FU (both curvilinear and linear trends had *p*s ≥ 0.59).

In contrast, there was no impact of the experimental manipulation on children's *internalizing* problems at T2 (*β* = −0.82, *p* = 0.63, *f* ^2^ < 0.01; *d* = −0.03), nor on the linear or curvilinear trends from T2 to T4 (both *p*s < 0.49; see Figure 2b). All estimated parameters can be found in Table 5 (model with linear trends only).

As a complement, the same model was re‐examined twice, positioning the intercept at T3 (6‐month FU) and T4 (1‐year FU) to document mean differences in externalizing and internalizing problems across both conditions at each of the FU assessments (see Tables S2 and S3, respectively). The impact of the experimental manipulation observed at T2 was still significant at T3 but became non‐significant at T4.

#### 4.2.2 Moderation

We explored whether parents' gender and family composition, as well as children's sex, age, and baseline level of behavior problems moderated the program's impact on child mental health at T2. Out of these five potential effect modifiers, none were significant, all *p*s ≥ 0.22 (Tables S4–S8).

#### 4.2.3 Within‐Condition Changes

Examining children's externalizing problems (see Figure 2a), parents in the How‐to condition (*β* = −9.40, *p* < 0.001; *d* = −0.54), as well as parents in the WL control condition (*β* = −6.62, *p* < 0.001; *d* = −0.29), reported a decrease from pre‐ to post‐intervention. These improvements were maintained over time (i.e., T1 to 6‐month FU; T1 to 1‐year FU) in the How‐to (all β*s* ≤ −10.80, all *p*s ≤ 0.001) and the WL condition (all *βs* ≤ −8.63, all *p*s ≤ 0.001).

Children's internalizing problems (see Figure 2b) also decreased from pre‐ to post‐intervention within both conditions (*β*_How‐to_ = −8.73, *p* < 0.001; *d* = −0.44; *β*_WL_ = −7.74, *p* < 0.001; *d* = −0.40). These differences were sustained over time within each condition (all *β*s in How‐to ≤ −9.82, all *p*s ≤ 0.001; all *β*s in WL ≤ −8.01, all *p*s ≤ 0.001).

### 4.3 Supplemental Analyses

#### 4.3.1 Mitigating Intervention Diffusion

Including waitlist parents who reported reading at least some of the How‐to book may have underestimated the sizes of the effects found. We thus re‐ran the same models after removing the 20 parents of the WL control condition who reported reading at least some of the How‐to book (see Table S9). The condition effect sizes for externalizing problems increased from *f* ^2^ 0.02 to 0.03 (*d* from 0.21 to 0.33) after conducting these sensitivity analyses.

#### 4.3.2 Practical Significance

The effect of our experimental manipulation was of small size, statistically. To help appreciate its practical meaning, we provide some complementary information. Though data for such analyses may be biased by missing data, they provide useful complementary information to get a practical sense of the changes reported. To assess whether the magnitude of change in externalizing behaviors is reliable, we first calculated *reliable change* indicators using the Jacobson and Truax (1992) method, which determines whether a change is large enough to rule out measurement error. To meet this criterion, change has to exceed a threshold expected for 95% of cases, which is based on the measure's reliability and the sample's pre‐intervention variability. Results showed that 29 children (19.7%) in the How‐to condition improved reliably, compared to 21 children (14.3%) in the WL one.

Next, it can be informative to examine the rates of children classified as falling above Borderline clinical cut‐offs at each time point, in each condition. We thus report the number of children whose externalizing score (raw sum scores) fell in the Borderline range of mental health problems, using CBCL normative data (*T*‐scores, based on age and sex; Achenbach and Rescorla 2001). At T1, about 37% of children's scores on externalizing problems fell in the Borderline range (36.9% and 37.4% in the experimental and WL conditions, respectively; see Table 4). Among those whose parents provided data at T2, the percentage of children whose score fell in that range was 16.7% in the How‐to condition, compared to 23.7% in the WL condition. At the 1‐year FU, 17.2% of children in the How‐to condition were in that range (vs. 21.7% in the WL condition).

## 5 Discussion

The present study used a waitlist RCT design to assess the impact of the How‐to Parenting Program on school‐aged children's mental health. Multivariate multilevel analyses indicated that compared to children whose parents were assigned to a waitlist, children whose parents were offered the How‐to Parenting Program displayed fewer externalizing problems over at least 6 months, according to their parents. This is noteworthy, as the stability of programs' effects on child mental health is not consistently found, even among selective and indicated efforts (Smedler et al. 2015). However, there were similar reductions in child internalizing problems across both conditions.

### 5.1 How‐to Parenting Program's Main Effects on Child Mental Health Problems

In a previous pre‐post study assessing the impact of the How‐to Parenting Program on children's mental health, both internalizing and externalizing problems were found to decrease (Joussemet et al. 2014), and these improvements, of large size, were found to be maintained over the following year (Mageau, Joussemet, Paquin, et al. 2022). In the present RCT, parents of both the experimental and the waitlist conditions reported that their child's problems decreased from pre‐ to post‐intervention before reaching a plateau. The pre‐post improvement within the waitlist control condition was unanticipated. Among the possible factors that may have contributed to it, there are possible efforts invested by waitlist parents, some learning derived from the questionnaires' items about parenting skills, maturation, spontaneous progress, and/or some exposure to the program's content via independent reading of the How‐to book. While no waitlist parent could take part in the program, not offered in the city where the study took place, it was possible to get access to the book, and perhaps more than 20 waitlist parents did so, as suggested by the elevated rate of waitlist parents skipping the reading question. Such diffusion of intervention may have led to an underestimation of the size of effects, as suggested by the larger effect size observed in the supplemental analyses conducted without waitlist parents reporting some reading. It is, unfortunately, impossible to know what the effect sizes would have been if the program's name had been concealed during information sessions, which we recommend for future trials. For the time being, the results obtained from the present RCT suggest that the How‐to Parenting Program can help decrease externalizing problems compared to our waitlist condition, not internalizing problems.

The lack of effect on internalizing problems in the present RCT is similar to what most universal parenting program evaluations report. In their meta‐analysis, Yap et al. (2016) reported that universal parenting interventions aiming to decrease child internalizing problems did not differ from control conditions (*d* = 0.11). A contamination‐free trial of the How‐to Parenting Program is needed to draw some conclusions about its impact on children's internalizing difficulties. In future studies, child‐reported measures could also be included for children who are old enough to complete them, as internalizing difficulties are less salient and child reports differ from parental ratings (De Los Reyes and Kazdin 2005).

To adequately interpret the externalizing effect found in the present RCT and fully appreciate its significance, it is informative to compare it to previous trials that have used a similar design (universal, intent‐to‐treat, waitlist‐RCT approach) and measure (CBCL). Unfortunately, we could only find one such study, which found no impact on school‐aged children's behavior problems (Malti et al. 2011). In a similar study, but targeting parents of 3‐ to 6‐year‐olds, results showed that the evaluated program had a positive impact in reducing preschoolers' problems, but only when they were rated by mothers of two‐parent families, not when they were rated by fathers or by single mothers (Hahlweg et al. 2010). The small but statistically significant effect of the How‐to Parenting Program on externalizing behaviors thus seems promising. Among children whose parents were assigned to the How‐to Parenting Program condition, a fifth improved reliably, and the percentage of children whose score fell in the Borderline range for externalizing problems decreased by 20% a year after program delivery (from 37% to 17%).

These findings are all the more encouraging considering that (1) universal preventive approaches often fail to have an impact on children's behaviors (Leijten et al. 2019), as floor effects are probably operating; (2) the stringent intent‐to‐treat analytical approach retained all participants (i.e., How‐to parents with low or no attendance and WL parents reporting some How‐to book reading); and (3) the How‐to Parenting Program first and foremost focuses on parents' practices (proximal outcomes) rather than on children's disruptive behaviors (distal outcomes).

The positive impact of the program on child externalizing problems may be there, not despite, but *because* of its focus on parenting quality. The program's content may indeed be responsible, at least partially, for the program's impact. Recently, the How‐to Parenting Program was found to foster favorable parenting practices (Mageau, Joussemet, Robichaud, et al. 2022). The present study complements that previous one by assessing whether this program also leads to better child mental health, the less proximal yet ultimate goal.

### 5.2 Lack of Moderation

The positive effect of the How‐to Parenting Program on child externalizing problems and the lack of effect on internalizing ones were not gender‐specific, nor restricted to a certain period during middle childhood. The documented impacts (or lack thereof) were thus similar for boys and girls, mothers and fathers, and across the ages of school‐aged children. They were not moderated by children's initial level of mental health problems either, suggesting that the program's benefits did not depend on children's initial difficulties. This latter result is discrepant from past studies reporting larger program effects for children experiencing greater difficulties (Bodenmann et al. 2008; Leijten et al. 2019; Lindsay and Totsika 2017). Lastly, the pattern of effects did not vary according to family composition. This is noteworthy, as investing time and energy in a parenting program is probably more challenging for single parents, who seem to benefit as much from the How‐to Parenting Program as participants of two‐parent households.

### 5.3 Strengths, Limitations, and Future Studies

The main strength of this study is its RCT design, which included a control condition and two follow‐ups during the following year. The program was delivered with high fidelity (Lafontaine et al. 2025), allowing us to attribute the present effects to it (Dane and Schneider 1998). The sample was also relatively large, with very little attrition over the course of the trial, and a relatively large number of fathers were successfully recruited. The program's manualized format and facilitators' training are additional strengths, as they foster content fidelity, while the use of validated instruments to examine the program's impact can ease potential replications. Next, multivariate multilevel analyses allowed for a rigorous and sound examination of the data and offered key insights into differences between conditions and progress across time points. Finally, in contrast to many other parenting program evaluations, this RCT is independent, which reduces the bias inherent in trials conducted with the input of program developers (Malti et al. 2011).

This study also has limitations. First, the waitlist design prevented participants' blinding, as participants offered the parenting program were aware of being in the experimental condition. An active control condition would be an asset to avoid this caveat in future trials. Second, information about the program should not have been revealed during information sessions, as it probably contributed to intervention diffusion across conditions. Indeed, though no parent in the control condition could take part in the program, they did learn about its name and could thus independently order its book while waiting for the program, which hindered differentiation. The effect sizes obtained in this trial may have been underestimated due to this diffusion of the intervention. Third, we solely relied on parent reports for assessments of child mental health problems. Although this limitation is the same across conditions, parents in the experimental condition may have been more tempted to report improvements, given the time and energy they had invested in taking part in a program. However, it seems unlikely that this bias fully explains the program's impact, as it was maintained for at least 6 months. Fourth, the sample was relatively well‐educated, suggesting a self‐selection bias, which could hinder the generalization of our results to the full population. Finally, the lack of moderation reported here should be interpreted cautiously and ideally replicated in larger samples.

Future studies are needed to identify the mechanisms that may be involved in the How‐to Parenting Program's impact. The program may have lowered externalizing problems by improving parents' communication skills and parenting style. Other variables also deserve to be examined, such as social support and parents' well‐being. By identifying mechanisms of change, greater light can be shed on parenting programs' active ingredients.

Future efficacy trials could also test whether younger children could benefit from the How‐to Parenting Program as well. Cost‐effectiveness analyses of this program also seem warranted, as it is an accessible resource that is relatively inexpensive to deliver. Other recommendations are to include a reading condition in future trials (alongside an entire program and a control condition) and to assess positive indicators of mental health, such as socio‐emotional strengths, to provide a comprehensive portrayal of the program's impact. Lastly, given that the How‐to book has been translated into more than 30 languages, assessing its impact in other parts of the world would be of both theoretical and practical value.

### 5.4 Theoretical and Practical Implications

Replicating the preliminary pre‐post trial's results on children's externalizing problems (Joussemet et al. 2014; Mageau, Joussemet, Paquin, et al. 2022), the present results suggest that delivering the How‐to Parenting Program is helpful to promote children's mental health when adopting a universal approach. The present study can be seen as contributing to the vast yet mainly correlational literature showing that parenting quality can foster child mental health. Further evaluating promising universal parenting programs seems crucial, as well as exploring the extent to which parental progress in AS, structure, and affiliation each represents a mechanism of change in the promotion of healthy child development and the prevention of psychopathology.

The present research indicates that the How‐to Parenting Program effectively decreased school‐aged children's externalized problems. Some improvements within 2 months can be quite encouraging for family members, potentially breaking the spiral of negative parent–child interactions and preventing the onset or worsening of child behavior problems in some families. Moreover, small changes can be meaningful for parents and encourage those whose children experience some lasting problems to seek additional help and not feel stigmatized by doing so. In that sense, efficacious universal programs can serve as a gateway for more targeted interventions. Research has shown that embedding targeted prevention programs within universal programs can produce synergistic effects (Dodge 2020).

Though statistically, the size of the How‐to Parenting Program's effect on externalizing difficulties was small, its presence and relative sustainability in a universal prevention context are encouraging, as a simple intervention offered to many can have a large preventive effect (Salari and Enebrink 2018). Indeed, offering this program in a universal way can lead to small mental health improvements at the individual level while representing an appreciable change when transposed at a population level. By teaching concrete skills, this strengths‐based program may help meet the needs of families in community settings and be a judicious prevention strategy investment in terms of public health.

## Conflicts of Interest

The authors declare no conflicts of interest.

## Supporting information


**Data S1:** famp70081‐sup‐0001‐TablesS1.docx.

## Data Availability

The data that support the findings of this study are available on reasonable request from the corresponding author. The data are not publicly available due to privacy or ethical restrictions.

## Endnotes

In the other studies, the ranges of children's ages were wider. When also including preschoolers, positive effects were perceived, but not by all respondents (Bodenmann et al. 2008; Hahlweg et al. 2010) or not at all time points (Reedtz et al. 2011). Among studies also including early adolescents, one program led to improvements (Havighurst et al. 2015), whereas the other did not.
