# Supplementary material for: Evaluating the Impact of the How‐to Parenting Program on Child Mental Health: A Randomized Controlled Trial in Grade Schools
Source: Fam Process. 2025 Nov 2;64(4):e70081. doi: 10.1111/famp.70081 (PMC12580021; doi:10.1111/famp.70081)
Supplement: Supplementary file 1 — Data S1: famp70081‐sup‐0001‐TablesS1.docx. [file FAMP-64-0-s001.docx]

# **Supplemental Material**

# **Table S1**

*Missing Data Pattern for the Full Study Sample Across Timepoints*

| Variables | Number of respondents | Missing data (%) |
| --- | --- | --- |
| **Categorical variables** |  |  |
| Child sex | 290 | 1.02 |
| Parent gender | 293 | 0.00 |
| Family income | 288 | 1.71 |
| Education | 285 | 2.73 |
| Family composition | 287 | 2.39 |
| **Continuous variables** |  |  |
| Child age | 291 | 0.68 |
| Parent age | 285 | 2.73 |
| AT parenting (CR) | 108 | 63.14 |
| AS parenting (PR) | 289 | 1.36 |
| T1 Externalizing behaviors | 289 | 1.36 |
| T1 Internalizing behaviors | 289 | 1.36 |
| T2 Externalizing behaviors | 242 | 17.41 |
| T2 Internalizing behaviors | 242 | 17.41 |
| T3 Externalizing behaviors | 245 | 16.38 |
| T3 Internalizing behaviors | 244 | 16.72 |
| T4 Externalizing behaviors | 238 | 18.77 |
| T4 Internalizing behaviors | 238 | 18.77 |

*Note*. AS = autonomy-supportive; AT = autonomy-thwarting; CR = child-reported; PR = parent-reported; T1 = pre-intervention; T2 = post-intervention; T3 = 6-month follow-up; T4 = 1-year follow-up. Only children aged 8 years or more were invited to report on the covariate AT parenting.

# **Table S2**

# *Multilevel Linear Model Testing the Impact of the Experimental Manipulation on Children’s Problem Behaviors – Intercept at the 6-month Follow-up (T3)*

| **Externalizing behaviors** | *β* | *SE* | *p* |
| --- | --- | --- | --- |
| Intercept (T3) | 22.02 | 0.94 | <0.01 |
| Experimental Condition^1^ | -2.97 | 1.48 | 0.04 |
| Parent age | 0.02 | 0.12 | 0.85 |
| Family income | -0.23 | 0.60 | 0.71 |
| T1 AT parenting (CR) | 0.79 | 2.32 | 0.74 |
| T1 AS parenting (PR) | 0.37 | 0.99 | 0.71 |
| T1 Externalizing behaviors | 0.58 | 0.03 | <0.01 |
| Slope (from T2 to T4) | -0.17 | 0.10 | 0.08 |
| Experimental Condition^1^ | 0.06 | 0.15 | 0.70 |
| Parent age | 0.01 | 0.01 | 0.40 |
| Family income | -0.01 | 0.05 | 0.82 |
| T1 AT parenting (CR) | -0.01 | 0.16 | 0.95 |
| T1 AS parenting (PR) | -0.09 | 0.10 | 0.36 |
| T1 Externalizing behaviors | -0.01 | 0.03 | <0.01 |
| **Internalizing behaviors** | *β* | *SE* | *p* |
| Intercept (T3) | 19.84 | 1.12 | <0.01 |
| Experimental Condition^1^ | -1.30 | 1.66 | 0.44 |
| Parent age | -0.05 | 0.14 | 0.75 |
| Family income | -0.11 | 0.58 | 0.85 |
| T1 AT parenting (CR) | -0.53 | 2.56 | 0.84 |
| T1 AS parenting (PR) | 0.6 | 1.17 | 0.61 |
| T1 Internalizing behaviors | 0.49 | 0.04 | <0.01 |
| Slope (from T2 to T4) | -0.01 | 0.11 | 0.98 |
| Experimental Condition^1^ | -0.08 | 0.17 | 0.64 |
| Parent age | -0.01 | 0.01 | 0.73 |
| Family income | -0.01 | 0.06 | 0.91 |
| T1 AT parenting (CR) | 0.08 | 0.17 | 0.64 |
| T1 AS parenting (PR) | -0.09 | 0.10 | 0.42 |
| T1 Internalizing behaviors | -0.11 | 0.01 | <0.01 |

*Note*. ^1^ Waitlist control condition [0] vs. How-to condition [1]; T1 = pre-intervention; T2 = post-intervention; T3 = 6-month follow-up; T4 = 1-year follow-up; AS = autonomy-supportive; AT = autonomy-thwarting; CR = child-reported; PR = parent-reported.

# **Table S3**

# *Multilevel Linear Model Testing the Impact of the Experimental Manipulation on Children’s Problem Behaviors – Intercept at the 1-year Follow-up (T4)*

| **Externalizing behaviors** | *β* | *SE* | *p* |
| --- | --- | --- | --- |
| Intercept (T4) | 20.99 | 1.12 | <0.01 |
| Experimental Condition^1^ | -2.60 | 1.90 | 0.17 |
| Parent age | 0.09 | 0.15 | 0.56 |
| Family income | -0.30 | 0.70 | 0.67 |
| T1 AT parenting (CR) | 0.72 | 2.63 | 0.78 |
| T1 AS parenting (PR) | -0.92 | 1.20 | 0.44 |
| T1 Externalizing behaviors | 0.51 | 0.04 | <0.01 |
| Slope (from T2 to T4) | -0.17 | 0.10 | 0.08 |
| Experimental Condition^1^ | 0.06 | 0.15 | 0.70 |
| Parent age | 0.01 | 0.01 | 0.40 |
| Family income | -0.01 | 0.05 | 0.82 |
| T1 AT parenting (CR) | -0.01 | 0.16 | 0.95 |
| T1 AS parenting (PR) | -0.09 | 0.10 | 0.36 |
| T1 Externalizing behaviors | -0.01 | 0.01 | <0.01 |
| **Internalizing behaviors** | *β* | *SE* | *p* |
| Intercept (T4) | 19.85 | 1.39 | <0.01 |
| Experimental Condition^1^ | -1.77 | 2.14 | 0.41 |
| Parent age | -0.75 | 0.17 | 0.66 |
| Family income | 0.15 | 0.72 | 0.83 |
| T1 AT parenting (CR) | -0.04 | 2.63 | 0.99 |
| T1 AS parenting (PR) | 0.09 | 1.33 | 0.95 |
| T1 Internalizing behaviors | 0.43 | 0.04 | <0.01 |
| Slope (from T2 to T4) | -0.01 | 0.11 | 0.98 |
| Experimental Condition^1^ | -0.08 | 0.17 | 0.64 |
| Parent age | -0.01 | 0.01 | 0.73 |
| Family income | -0.01 | 0.06 | 0.91 |
| T1 AT parenting (CR) | 0.08 | 0.17 | 0.64 |
| T1 AS parenting (PR) | -0.09 | 0.10 | 0.42 |
| T1 Internalizing behaviors | -0.11 | 0.02 | <0.01 |

*Note*. ^1^ Waitlist control condition [0] vs. How-to condition [1]; T1 = pre-intervention; T2 = post-intervention; T3 = 6-month follow-up; T4 = 1-year follow-up; AS = autonomy-supportive; AT = autonomy-thwarting; CR = child-reported; PR = parent-reported.

# **Table S4**

# *Multilevel Linear Model Testing the Impact of the Experimental Manipulation on Children’s Problem Behaviors – Interaction with Child Age*

| **Externalizing behaviors** | *β* | *SE* | *p* |
| --- | --- | --- | --- |
| Intercept (T2) | 23.05 | 1.10 | <0.01 |
| Experimental Condition^1^ | -3.30 | 1.57 | 0.04 |
| Parent age | -0.05 | 0.14 | 0.71 |
| Family income | -0.09 | 0.62 | 0.89 |
| T1 AT parenting (CR) | 1.21 | 2.43 | 0.61 |
| T1 AS parenting (PR) | 0.14 | 1.10 | 0.90 |
| T1 Externalizing behaviors | 0.64 | 0.04 | <0.01 |
| Child age | 0.42 | 0.50 | 0.40 |
| Child age * Condition^1^ | -0.85 | 0.76 | 0.26 |
| Slope (from T2 to T4) | -0.17 | 0.10 | 0.07 |
| Experimental Condition^1^ | 0.07 | 0.15 | 0.65 |
| Parent age | 0.01 | 0.01 | 0.34 |
| Family income | -0.01 | 0.05 | 0.82 |
| T1 AT parenting (CR) | -0.02 | 0.16 | 0.91 |
| T1 AS parenting (PR) | -0.09 | 0.10 | 0.35 |
| T1 Externalizing behaviors | -0.11 | 0.01 | <0.01 |
| Child age | -0.03 | 0.05 | 0.51 |
| Child age * Condition^1^ | 0.02 | 0.08 | 0.81 |
| **Internalizing behaviors** | *β* | *SE* | *p* |
| Intercept (T2) | 19.80 | 1.17 | <0.01 |
| Experimental Condition^1^ | -0.77 | 1.72 | 0.66 |
| Parent age | -0.02 | 0.16 | 0.89 |
| Family income | -0.01 | 0.64 | 0.99 |
| T1 AT parenting (CR) | -0.73 | 2.98 | 0.81 |
| T1 AS parenting (PR) | 1.08 | 1.32 | 0.41 |
| T1 Internalizing behaviors | 0.56 | 0.04 | <0.01 |
| Child age | 0.34 | 0.61 | 0.58 |
| Child age * Condition^1^ | -0.74 | 0.93 | 0.43 |
| Slope (from T2 to T4) | 0.01 | 0.11 | 0.93 |
| Experimental Condition^1^ | -0.09 | 0.17 | 0.58 |
| Parent age | -0.01 | 0.01 | 0.59 |
| Family income | -0.01 | 0.06 | 0.94 |
| T1 AT parenting (CR) | 0.09 | 0.18 | 0.59 |
| T1 AS parenting (PR) | -0.09 | 0.11 | 0.40 |
| T1 Internalizing behaviors | -0.11 | 0.03 | <0.01 |
| Child age | 0.04 | 0.06 | 0.48 |
| Child age * Condition^1^ | -0.02 | 0.10 | 0.81 |

*Note*. ^1^ Waitlist control condition [0] vs. How-to condition [1]; T1 = pre-intervention; T2 = post-intervention; T3 = 6-month follow-up; T4 = 1-year follow-up; AS = autonomy-supportive; AT = autonomy-thwarting; CR = child-reported; PR = parent-reported.

# **Table S5**

# *Multilevel Linear Model Testing the Impact of the Experimental Manipulation on Children’s Problem Behaviors – Interaction with Parental Gender*

| **Externalizing behaviors** | *β* | *SE* | *p* |
| --- | --- | --- | --- |
| Intercept (T2) | 22.88 | 1.16 | <0.01 |
| Experimental Condition^1^ | -3.18 | 1.71 | 0.06 |
| Parent age | -0.05 | 0.15 | 0.74 |
| Family income | -0.18 | 0.63 | 0.78 |
| T1 AT parenting (CR) | 0.86 | 2.39 | 0.72 |
| T1 AS parenting (PR) | 0.24 | 1.08 | 0.82 |
| T1 Externalizing behaviors | 0.64 | 0.03 | <0.01 |
| Parental gender^2^ | 0.98 | 3.00 | 0.75 |
| Parental gender^2^ * Condition^11^ | -0.84 | 3.97 | 0.83 |
| Slope (from T2 to T4) | -0.16 | 0.10 | 0.12 |
| Experimental Condition | 0.08 | 0.16 | 0.60 |
| Parent age | 0.01 | 0.01 | 0.36 |
| Family income | -0.01 | 0.05 | 0.85 |
| T1 AT parenting (CR) | -0.03 | 0.16 | 0.85 |
| T1 AS parenting (PR) | -0.10 | 0.10 | 0.32 |
| T1 Externalizing behaviors | -0.01 | 0.01 | <0.01 |
| Parental gender^2^ | -0.08 | 0.27 | 0.77 |
| Parental gender^2^ * Condition^1^ | -0.09 | 0.39 | 0.82 |
| **Internalizing behaviors** | *β* | *SE* | *p* |
| Intercept (T2) | 19.36 | 1.21 | <0.01 |
| Experimental Condition^1^ | -0.93 | 1.84 | 0.61 |
| Parent age | -0.05 | 0.17 | 0.78 |
| Family income | -0.12 | 0.63 | 0.85 |
| T1 AT parenting (CR) | -0.72 | 2.85 | 0.80 |
| T1 AS parenting (PR) | 1.33 | 1.29 | 0.30 |
| T1 Internalizing behaviors | 0.56 | 0.04 | <0.01 |
| Parental gender^2^ | 2.95 | 3.94 | 0.46 |
| Parental gender^2^ * Condition^1^ | -0.50 | 4.81 | 0.92 |
| Slope (from T2 to T4) | 0.02 | 0.12 | 0.90 |
| Experimental Condition^1^ | -0.05 | 0.18 | 0.77 |
| Parent age | -0.01 | 0.01 | 0.81 |
| Family income | -0.01 | 0.06 | 0.92 |
| T1 AT parenting (CR) | 0.06 | 0.18 | 0.73 |
| T1 AS parenting (PR) | -0.09 | 0.10 | 0.37 |
| T1 Internalizing behaviors | -0.01 | <0.01 | <0.01 |
| Parental gender^2^ | -0.09 | 0.27 | 0.73 |
| Parental gender^2^ * Condition^1^ | -0.07 | 0.42 | 0.86 |

*Note*. ^1^ Waitlist control condition [0] vs. How-to condition [1]; ^2^ Mother = [0] vs. Father [1]; T1 = pre-intervention; T2 = post-intervention; T3 = 6-month follow-up; T4 = 1-year follow-up; AS = autonomy-supportive; AT = autonomy-thwarting; CR = child-reported; PR = parent-reported.

**Table S6**

*Multilevel Linear Model Testing the Impact of the Experimental Manipulation on Children’s Problem Behaviors – Interaction with (T1) Child Problem Behaviors*

| **Externalizing behaviors** | *β* | *SE* | *p* |
| --- | --- | --- | --- |
| Intercept (T2) | 22.96 | 1.10 | <0.01 |
| Experimental Condition^1^ | -3.30 | 1.56 | 0.03 |
| Parent age | -0.04 | 0.14 | 0.77 |
| Family income | -0.16 | 0.63 | 0.80 |
| T1 AT parenting (CR) | 1.41 | 2.68 | 0.60 |
| T1 AS parenting (PR) | 0.23 | 1.11 | 0.84 |
| T1 Externalizing behaviors | 0.65 | 0.05 | <0.01 |
| T1 Externalizing behaviors * Condition^1^ | -0.02 | 0.07 | 0.79 |
| Slope (from T2 to T4) | -0.17 | 0.10 | 0.08 |
| Experimental Condition^1^ | 0.05 | 0.15 | 0.76 |
| Parent age | 0.01 | 0.01 | 0.40 |
| Family income | -0.01 | 0.05 | 0.81 |
| T1 AT parenting (CR) | 0.03 | 0.19 | 0.89 |
| T1 AS parenting (PR) | -0.08 | 0.10 | 0.40 |
| T1 Externalizing behaviors | -0.01 | <0.01 | 0.07 |
| T1 Externalizing behaviors * Condition^1^ | -0.01 | <0.01 | 0.55 |
| **Internalizing behaviors** | *β* | *SE* | *p* |
| Intercept (T2) | 20.08 | 1.20 | <0.01 |
| Experimental Condition^1^ | -0.72 | 1.75 | 0.68 |
| Parent age | -0.02 | 0.16 | 0.91 |
| Family income | -0.05 | 0.63 | 0.94 |
| T1 AT parenting (CR) | -0.83 | 2.51 | 0.74 |
| T1 AS parenting (PR) | 0.98 | 1.31 | 0.46 |
| T1 Internalizing behaviors | 0.52 | 0.01 | <0.01 |
| T1 Internalizing behaviors * Condition^1^ | 0.05 | 0.01 | 0.50 |
| Slope (from T2 to T4) | -0.01 | 0.11 | 0.98 |
| Experimental Condition^1^ | -0.10 | 0.17 | 0.56 |
| Parent age | -0.01 | 0.01 | 0.77 |
| Family income | -0.01 | 0.06 | 0.90 |
| T1 AT parenting (CR) | 0.14 | 0.26 | 0.60 |
| T1 AS parenting (PR) | -0.07 | 0.10 | 0.48 |
| T1 Internalizing behaviors | -0.01 | 0.01 | 0.01 |
| T1 Internalizing behaviors * Condition^1^ | -0.004 | 0.005 | 0.46 |

*Note*. ^1^ Waitlist control condition [0] vs. How-to condition [1]; T1 = pre-intervention; T2 = post-intervention; T3 = 6-month follow-up; T4 = 1-year follow-up; AS = autonomy-supportive; AT = autonomy-thwarting; CR = child-reported; PR = parent-reported.

# **Table S7**

# *Multilevel Linear Model Testing the Impact of the Experimental Manipulation on Children’s Problem Behaviors – Interaction with Child Sex*

| **Externalizing behaviors** | *β* | *SE* | *p* |
| --- | --- | --- | --- |
| Intercept (T2) | 22.81 | 1.49 | < 0.01 |
| Experimental Condition^1^ | -4.75 | 2.08 | 0.02 |
| Parent age | -0.03 | 0.14 | 0.81 |
| Family income | -0.15 | 0.64 | 0.82 |
| T1 AT parenting (CR) | 0.54 | 2.27 | 0.81 |
| T1 AS parenting (PR) | 0.12 | 1.10 | 0.91 |
| T1 Externalizing behaviors | 0.64 | 0.04 | <0.01 |
| Child sex^2^ | 0.39 | 2.16 | 0.85 |
| Child sex^2^ * Condition^1^ | 3.04 | 3.07 | 0.32 |
| Slope (from T2 to T4) | -0.15 | 0.14 | 0.29 |
| Experimental Condition^1^ | 0.06 | 0.22 | 0.79 |
| Parent age | 0.01 | 0.01 | 0.42 |
| Family income | -0.01 | 0.05 | 0.81 |
| T1 AT parenting (CR) | -0.01 | 0.16 | 0.99 |
| T1 AS parenting (PR) | -0.09 | 0.10 | 0.36 |
| T1 Externalizing behaviors | -0.01 | 0.003 | <0.01 |
| Child sex^2^ | -0.04 | 0.20 | 0.85 |
| Child sex^2^ * Condition^1^ | -0.01 | 0.29 | 0.97 |
| **Internalizing behaviors** | *β* | *SE* | *p* |
| Intercept (T2) | 20.04 | 1.62 | <0.01 |
| Experimental Condition^1^ | -2.75 | 2.15 | 0.20 |
| Parent Age | -0.01 | 0.16 | 0.95 |
| Family income | -0.04 | 0.63 | 0.95 |
| T1 AT parenting (CR) | -1.19 | 2.79 | 0.67 |
| T1 AS parenting (PR) | 1.08 | 1.33 | 0.41 |
| T1 Internalizing behaviors | 0.56 | 0.04 | <0.01 |
| Child sex^2^ | -0.53 | 2.30 | 0.82 |
| Child sex^2^ * Condition^1^ | 4.02 | 3.41 | 0.24 |
| Slope (from T2 to T4) | -0.10 | 0.15 | 0.52 |
| Experimental Condition^1^ | 0.05 | 0.23 | 0.81 |
| Parent Age | -0.01 | 0.01 | 0.73 |
| Family income | -0.01 | 0.06 | 0.86 |
| T1 AT parenting (CR) | 0.07 | 0.17 | 0.70 |
| T1 AS parenting (PR) | -0.09 | 0.11 | 0.38 |
| T1 Internalizing behaviors | -0.01 | 0.003 | <0.01 |
| Child sex^2^ | 0.20 | 0.21 | 0.34 |
| Child sex^2^ * Condition^1^ | -0.27 | 0.31 | 0.39 |

*Note*. ^1^ Waitlist control condition [0] vs. How-to condition [1]; ^2^ Girl [0] vs. Boy [1]; T1 = pre-intervention; T2 = post-intervention; T3 = 6-month follow-up; T4 = 1-year follow-up; AS = autonomy-supportive; AT = autonomy-thwarting; CR = child-reported; PR = parent-reported.

# **Table S8**

# *Multilevel Linear Model Testing the Impact of the Experimental Manipulation on Children’s Problem Behaviors – Interaction with Family Composition (One-parent vs. Two-parent Family)*

| **Externalizing behaviors** | *β* | *SE* | *p* |
| --- | --- | --- | --- |
| Intercept (T2) | 23.13 | 1.18 | <0.01 |
| Experimental Condition^1^ | -3.43 | 1.67 | 0.04 |
| Parent age | -0.05 | 0.15 | 0.76 |
| Family income | -0.18 | 0.63 | 0.77 |
| T1 AT parenting (CR) | 0.90 | 2.40 | 0.71 |
| T1 AS parenting (PR) | 0.21 | 1.11 | 0.85 |
| T1 Externalizing behaviors | 0.64 | 0.04 | <0.01 |
| Family composition^2^ | -0.77 | 2.85 | 0.79 |
| Family composition^2^ * Condition^1^ | 0.95 | 4.63 | 0.84 |
| Slope (from T2 to T4) | -0.19 | 0.11 | 0.08 |
| Experimental Condition^1^ | 0.01 | 0.16 | 0.93 |
| Parent age | 0.01 | 0.01 | 0.48 |
| Family income | 0.01 | 0.05 | 0.85 |
| T1 AT parenting (CR) | 0.00 | 0.16 | 0.99 |
| T1 AS parenting (PR) | -0.10 | 0.10 | 0.32 |
| T1 Externalizing behaviors | -0.01 | 0.003 | <0.01 |
| Family composition^2^ | 0.14 | 0.22 | 0.52 |
| Family composition^2^ * Condition^1^ | 0.21 | 0.38 | 0.59 |
| **Internalizing behaviors** | *β* | *SE* | *p* |
| Intercept (T2) | 19.52 | 1.22 | <0.01 |
| Experimental Condition^1^ | -0.04 | 1.84 | 0.98 |
| Parent age | 0.00 | 0.16 | 0.99 |
| Family income | -0.09 | 0.63 | 0.89 |
| T1 AT parenting (CR) | -1.20 | 2.83 | 0.67 |
| T1 AS parenting (PR) | 1.03 | 1.28 | 0.42 |
| T1 Internalizing behaviors | 0.57 | 0.05 | <0.01 |
| Family composition^2^ | 2.42 | 3.35 | 0.47 |
| Family composition^2^ * Condition^1^ | -5.37 | 4.96 | 0.28 |
| Slope (from T2 to T4) | 0.01 | 0.11 | 0.92 |
| Experimental Condition^1^ | -0.08 | 0.18 | 0.66 |
| Parent age | -0.01 | 0.01 | 0.73 |
| Family income | -0.01 | 0.05 | 0.83 |
| T1 AT parenting (CR) | 0.08 | 0.17 | 0.63 |
| T1 AS parenting (PR) | -0.08 | 0.10 | 0.42 |
| T1 Internalizing behaviors | -0.01 | 0.003 | <0.01 |
| Family composition^2^ | -0.06 | 0.34 | 0.85 |
| Family composition^2^ * Condition^1^ | 0.02 | 0.52 | 0.97 |

*Note*. ^1^ Waitlist control condition [0] vs. How-to condition [1]; ^2^ Two-parent family = [0] vs. One-parent family [1]; T1 = pre-intervention; T2 = post-intervention; T3 = 6-month follow-up; T4 = 1-year follow-up; AS = autonomy-supportive; AT = autonomy-thwarting; CR = child-reported; PR = parent-reported.

**Table S9**

*Multilevel Linear Model Testing the Impact of the Experimental Manipulation on Children’s Problem Behaviors – Without Waitlist Participants Reading the How-to Book*

| **Externalizing behaviors** | *β* | *SE* | *p* |
| --- | --- | --- | --- |
| Intercept (T2) | 24.01 | 1.25 | <0.01 |
| Experimental Condition^1^ | -4.77 | 1.68 | <0.01 |
| Parent age | -0.06 | 0.15 | 0.67 |
| Family income | -0.23 | 0.59 | 0.71 |
| T1 AT parenting (CR) | 0.95 | 2.60 | 0.72 |
| T1 AS parenting (PR) | 0.09 | 1.22 | 0.94 |
| T1 Externalizing behaviors | 0.64 | 0.03 | <0.01 |
| Slope (from T2 to T4) | -0.21 | 0.12 | 0.08 |
| Experimental Condition^1^ | 0.13 | 0.17 | 0.45 |
| Parent age | 0.01 | 0.01 | 0.39 |
| Family income | -0.01 | 0.06 | 0.88 |
| T1 AT parenting (CR) | -0.02 | 0.21 | 0.94 |
| T1 AS parenting (PR) | -0.09 | 0.12 | 0.45 |
| T1 Externalizing behaviors | -0.01 | 0.003 | <0.01 |
| **Internalizing behaviors** | *β* | *SE* | *p* |
| Intercept (T2) | 20.25 | 1.31 | <0.01 |
| Experimental Condition^1^ | -1.52 | 1.76 | 0.39 |
| Parent age | -0.03 | 0.16 | 0.86 |
| Family income | -0.09 | 0.64 | 0.88 |
| T1 AT parenting (CR) | -0.92 | 2.56 | 0.72 |
| T1 AS parenting (PR) | 1.08 | 1.30 | 0.41 |
| T1 Internalizing behaviors | 0.56 | 0.03 | <0.01 |
| Slope (from T2 to T4) | 0.02 | 0.13 | 0.90 |
| Experimental Condition^1^ | -0.10 | 0.17 | 0.56 |
| Parent age | -0.01 | 0.02 | 0.75 |
| Family income | -0.01 | 0.06 | 0.90 |
| T1 AT parenting (CR) | 0.08 | 0.21 | 0.70 |
| T1 AS parenting (PR) | -0.09 | 0.13 | 0.49 |
| T1 Internalizing behaviors | -0.01 | 0.003 | <0.01 |

*Note*. ^1^ Waitlist control condition [0] vs. How-to condition [1]; T1 = pre-intervention; T2 = post-intervention; T3 = 6-month follow-up; T4 = 1-year follow-up; AS = autonomy-supportive; AT = autonomy-thwarting; CR = child-reported; PR = parent-reported.
